# Strategies for improving access to primary care services for homeless immigrants in England: a Delphi study

**DOI:** 10.1017/S1463423623000646

**Published:** 2023-12-14

**Authors:** Carol Namata, Eleni Hatzidimitriadou

**Affiliations:** Faculty of Medicine, Health and Social Care, Canterbury Christ Church University, Canterbury, Kent, England

**Keywords:** access to healthcare, homeless immigrants, primary care services

## Abstract

**Aim:**

The aim of the study was to identify the most prioritized strategies in improving access to primary care services (PCS) for homeless immigrants.

**Background:**

The issue of improving access to PCS for homeless immigrants is a complex and multifaceted one, and yet there is limited research on the strategies aimed at improving these services. Hence, the need for more studies that directly engage homeless immigrants and service providers in understanding their barriers to accessing PCS and their preferences for improving access to these services.

**Methods:**

The study used a two round Delphi method to elicit the views of stakeholders. The Delphi process utilized a web-based questionnaire. The stakeholders included healthcare providers and voluntary sector providers. The first round had a total of 58 items belonging to 14 categories. The second round comprised a total of 25 items belonging to 12 categories which were preselected based on participants’ ranking of their importance in the first round. Participants were required to rank the relative importance of all the items on a 5-point Likert scale. Data were analysed using the STATA-15 software package.

**Findings:**

A total of 12 stakeholders participated in both rounds of the Delphi survey. The top three strategies encompassed fighting against discrimination and prejudice, improving and promoting mental health services, and empowering homeless immigrants. These evidence-based strategies hold the potential to support the implementation of healthcare interventions aimed at improving access to PCS and healthcare outcomes for homeless immigrants. However, it is crucial to conduct further research that includes homeless immigrants in the Delphi study to gain insights into the strategies that are most important to them in enhancing access to PCS, as they are the primary target users. Such research will contribute to the development of comprehensive and effective interventions tailored to the specific needs of homeless.

## Introduction

Over the past decade, the issue of immigration and its implications for immigrants, host countries, and countries of their origin has been of high importance for global health and public policy (Wickramage *et al.*, 2018). In the United Kingdom (UK), it is estimated that long-term immigration increased from 942 000 in 2021 to 1.2 million by the end of 2022 (Office for National Statistics, 2023), and over this duration, there has been a surge in the literature surrounding health and social status of immigrants in the UK. Immigrants often arrive in their host countries with poorer general health status such as poorer mental health status compared to their non-immigrant counterparts due to their traumatic experiences of violence, conflicts, forced migration at short notice, and living in refugee camps (Woodgate *et al.*, 2017). Being outside their country of nationality can contribute to difficulties related to cultural differences, limited health system literacy, socioeconomic disadvantage, fear of being persecuted, and fear of deportation (Farcas and Gonçalves, 2017; Segal, 2019; Charitonos *et al.*, 2020; Rowley *et al*., 2020). Such challenges can hinder immigrants’ access to health care services, including primary care services (PCS). Furthermore, research evidence from the UK and other settings shows that there are intersections between migration and homelessness, whereby immigrants and refugees with limited social support in their host country are at greater risk of experiencing homelessness (Fitzpatrick and Pleace, 2012; Hermans *et al.*, 2020).

In England, the number of migrants experiencing or at risk of homelessness is increasing, yet the crisis of migrant homelessness remains largely invisible. A report by Crisis on understanding migrant homelessness unveiled a concerning reality: nearly 67% of the 83 survey respondents reported an increase in migrant homelessness in the areas where they had worked in the last 12 months. Furthermore, when delving into specific migrant subgroups, 24% of the survey respondents revealed a substantial increase in homelessness over the same period for people with no recourse to public funds or irregular status (Boobis *et al*., 2019, p.7). Additionally, an analysis by Shelter indicated that no less than 39% of people acknowledge that residing in temporary accommodations has presented formidable barriers in terms of accessing essential healthcare appointments (Shelter, 2023).

Existing research on PCS for immigrants in the UK has identified numerous barriers such as language barriers coupled with inadequate interpretation services; financial hardship in accessing dental care, prescription fees, and transport to appointments; and the experience of discrimination relating to race, religion, and immigration status (Fang *et al.*, 2015; Kang *et al.*, 2019; Asif and Kienzler, 2022). Such challenges experienced in accessing PCS might force migrants (with or without legal status) to instead use emergency health services more compared to native populations, which can result in disruptions in the delivery of emergency services (Credé *et al.*, 2018). Although immigrants were less likely to use primary care than non-immigrants before the COVID-19 pandemic, research shows that the pandemic exacerbated this difference in England particularly among children, certain ethnic groups, and migrants whose first language was not English (Zhang *et al*., 2022). Homeless immigrants often experience additional challenges in accessing PCS such as living further than walking distance from their GP, discrimination, and victimization, and being unaware that they must register with a GP (Gunner *et al*., 2019).

Improved access to primary care is crucial to marginalized groups including homeless individuals, refugees, asylum seekers, and irregular migrants. This is because these groups have poorer health outcomes due to their lived experiences compared to their host population (Schouler-Ocak *et al.*, 2016). Additionally, the under-use of these services may have significant repercussions on public health. The delay to seek treatment can lead to the further spread of communicable diseases while untreated chronic conditions might deteriorate and lead to increased costs of treatment (Spitzer *et al.*, 2019). Being the first point of contact in the healthcare system and acting as the front door of the National Health Service (NHS) implies that PCS form the largest part of most people’s experiences of healthcare and thus it is imperative to improve the health of the population (Newell, 2016).

Improving access to PCS for homeless immigrants presents a complex and multifaceted challenge stemming from a combination of linguistic, cultural, bureaucratic, social, and healthcare-related challenges (Kang *et al.*, 2019). Recognizing and addressing these complexities are essential steps in ensuring equitable and effective healthcare for this vulnerable population. However, there is limited research on the experiences and perspectives of homeless immigrants themselves, especially during the COVID-19 pandemic when access to PCS may have been further contrained (Kang *et al*., 2019; Tomkow *et al*., 2020). Consequently, there is a need for further research that actively involves homeless immigrants and service providers to delve deeply into the intricate web of challenges faced by homeless immigrants when seeking access to PCS and uncover their preferences for improving access (Woodward *et al.*, 2014). In addition, researchers and policymakers focused on improving the health of disadvantaged groups should be aware that healthcare providers themselves may contribute to barriers in healthcare access. Acknowledging these provider experiences as a viable area for action underscores the potential for developing future policies and research endeavours that effectively tackle this issue (Rivenbark and Ichou, 2020). Furthermore, there is limited attention to the intersection of homelessness, immigration status, and healthcare access in the UK (Hanley *et al.*, 2019). Scholars have also recommended that research on immigrant health needs to utilize more holistic research approaches so as to provide richer insight on social determinants and intersectionality in immigrant health (Viruell-Fuentes *et al.*, 2012; Piacentini *et al.*, 2019). This would contribute to the development of interventions that are tailored to the specific needs and cultural backgrounds of homeless immigrants (Crawford *et al*., 2022).

In the present study, we employ a Delphi approach to systematically identify the top prioritized strategies needed to improve access to PCS among homeless immigrants. These strategies were derived from a list of suggestions and opinions raised by homeless immigrants and service providers in the broader study. However, only service providers or stakeholders participated in this Delphi study. The study aimed to accomplish two specific objectives: (i) to determine the relative importance of each of the suggestions/items identified from the initial stakeholder and homeless immigrant interviews on improving access to PCS for homeless immigrants and (ii) to identify the top ten most prioritized strategies in improving access to PCS for homeless immigrants.

## Methods

### Study design, site, and participants

This study was part of a larger study on access to PCS for immigrants experiencing homelessness in Southeast England. Briefly, this larger study was a qualitative study involving 30 homeless immigrants and 30 stakeholders with an overarching aim of understanding the factors that impact access to PCS for homeless immigrants. Of the 30 stakeholders who participated in the initial semi-structured interviews, 22 of them were approached in writing via email and invited to participate in the Delphi survey. Participants were ensured anonymity with respect to their opinions. All the participants were required to have working experience in migrant health or social issues and be actively involved in service provision at the time this study was being conducted. The stakeholders who were healthcare providers, voluntary sector providers, and local council professionals were purposely contacted from various organizations in Kent and London to ensure that different perspectives were represented (Thomas *et al.*, 2019).

### Study tool development

Initially, semi-structured interviews were conducted between November 2021 and May 2022 with homeless immigrants and stakeholders with the aim of identifying the factors that impact access to PCS for homeless immigrants. A list of suggestions/actions to improve access to PCS for homeless immigrants was identified through thematic analysis of the data from the semi-structured interviews. This list was used to inform the development of the Delphi survey. The Delphi method is widely recommended as a means for collecting and synthesizing expert opinion on a given issue in the field of their expertise (Devillé *et al*., 2011; Barrios *et al.*, 2021). The Delphi survey tool was vetted by a panel of experienced researchers and service providers involved in immigrant health to ascertain its content and face validity. The tool was pretested among two stakeholders who did not take part in the final survey.

### Data collection

This Delphi survey was administered in two rounds using the Jisc online survey platform (www.jisc.ac.uk) between December 2022 and April 2023.

The first round of the Delphi survey was conducted to facilitate consensus among stakeholders on the relative importance of strategies to improve access to PCS for homeless immigrants. An invitation with details of the study and a link to the online questionnaire was sent by email to the 22 stakeholders requesting them to participate in the first round of the Delphi Survey. This Delphi survey in the first round had a total of 58 items belonging to 14 categories (see Supplementary file 1). Participants were required to rank the relative importance of each of the 58 items on a 5-point Likert scale (1 = Not important at all, 2 = Not very important, 3 = Moderately important, 4 = Important 5 = Very important). At the end of each of the 14 categories, participants had the option of adding any further comments/reflections if applicable. On average, it took 20–30 min to complete the round one Delphi survey. Data collection for round one was conducted between December 2022 and January 2023. Participants who did not respond to the study invitation were sent a reminder after two, four, and six weeks from the initial invite.

The second round of the Delphi survey was conducted between February and April 2023. Participants were eligible to participate in round two of the Delphi survey if they had completed round one. Participants were invited to participate in the second round following similar steps described earlier for the first round. This second round of the Delphi survey comprised a reduced number of items which were preselected based on participants’ ranking of their importance in the first round as described in the analysis section. The ultimate aim of this second round was to identify the top 10 most prioritized items or suggestions for improving access to PCS. After two weeks, four weeks, and six weeks, non-respondents received a reminder. On average, it took 10–15 min to complete round two of the Delphi survey.

### Data processing and analysis

We used descriptive statistics including percentages and means with their standard deviations to summarize participants’ demographic characteristics and the results from rounds one and two of the Delphi survey.

In round one of the Delphi survey, the items which were ranked as *important* or *very important* by 75% or more of the respondents were considered to have achieved consensus. A 75% consensus has also been used in other Delphi studies involving health professionals (Orsini *et al.*, 2023). The items which did not reach consensus were excluded from the second round. Furthermore, we computed the mean Likert score of the items that achieved consensus and retained those with a mean score of 4.5 or higher to be included in the second round of the Delphi survey. A cut-off of mean or median scores of 4.5 on a 5-point Likert scale has been previously used in other health-related research studies (Hobbelen *et al.*, 2006).

**Figure 1.**
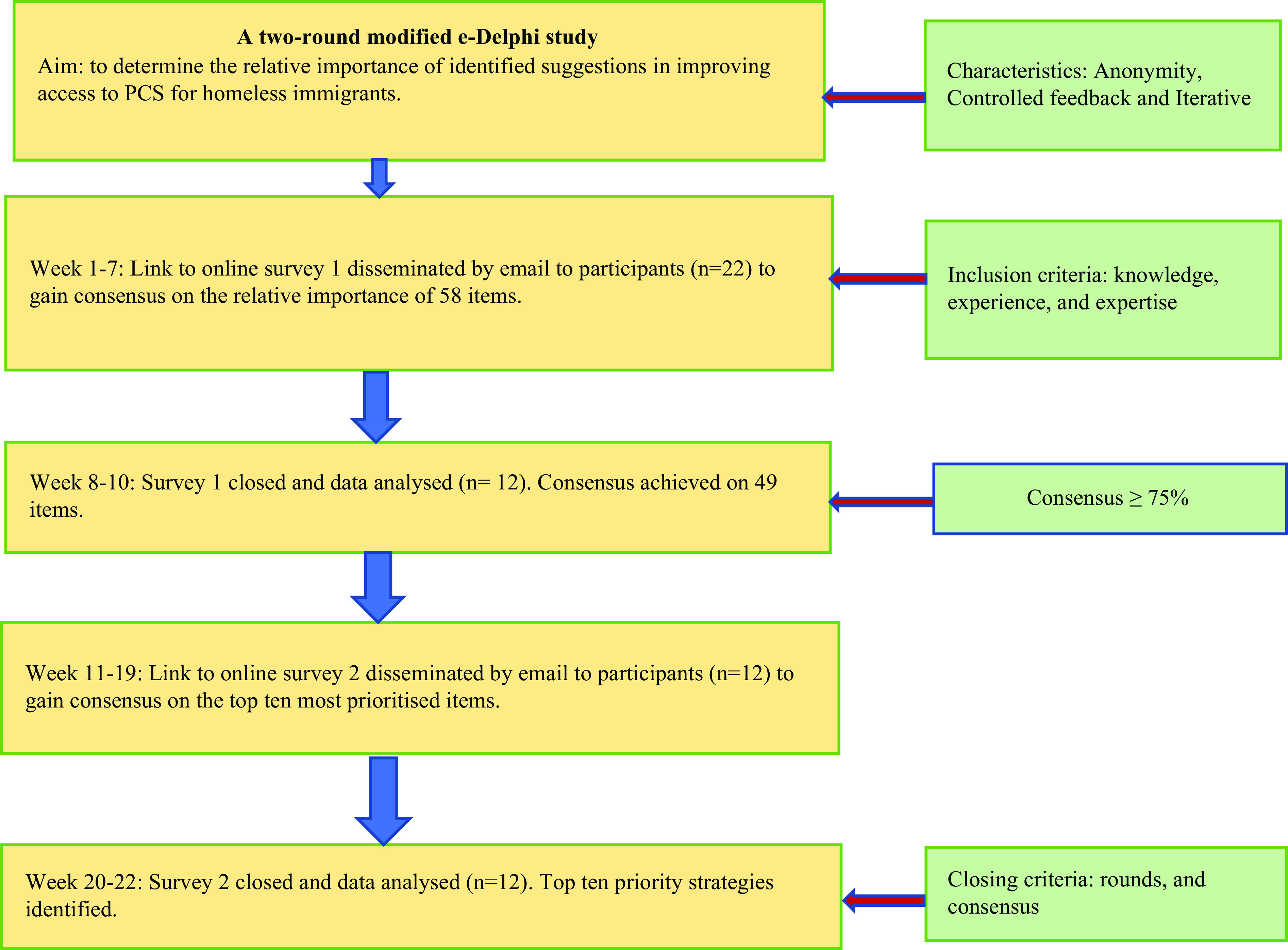
Flow diagram of the Delphi process (Currie *et al*., 2022)

In the final round (second round), we computed the mean scores and their standard deviation and selected 10 items which had the highest mean scores. These 10 items were ultimately considered as the top priority strategies identified by stakeholders for improving access to PCS for homeless immigrants. All the data analysis was conducted in STATA-15 software package. A flow diagram of the methods is shown in Figure 1.

### Ethical considerations

Prior to completing the Delphi survey, written informed consent was obtained from all study participants. The informed consent form was part of the online survey tool and had to be completed prior to accessing the rest of the survey material. Confidentiality and anonymity were ensured by using participants’ study codes as opposed to personal identifiers. This study was approved by the ethics review board of Canterbury Christ Church University (ETH2223-0076).

## Results

A total of 12 participants completed round one and the same number of participants completed round two of the Delphi survey, and their demographic characteristics are presented in Table 1. Most respondents provided services in the County of Kent. Most were females (75%) and healthcare providers with nurse practitioners being the majority (33%).

**Table 1.** Demographic characteristics of the Delphi panel (*n* = 12)

Variable	*n* (%)
**Sex**	
Male	3 (25)
Female	9 (75)
**Location**	
Kent	9(75)
London	3(25)
**Profession**	
Healthcare providers	
Nurse practitioners	4 (33.3)
Specialist caseworkers	2 (16.7)
General practitioner	1 (8.3)
Practice manager	1 (8.3)
**Voluntary sector providers**	
Project workers	2 (16.7)
Mentoring coordinators	2 (16.7)

In the first round, 49 of the 58 items reached 75% frequency or more in ranking as important or very important (i.e., achieved consensus) by stakeholders (see Supplementary file 1). The 9 items that did not achieve consensus belonged to the categories of; *improving communication between immigrants and healthcare providers* (*n* = 2); *improving the quality of PCS* (*n* = 2); *provision of culturally sensitive PCS* (*n* = 1); *improving and promoting mental health services among homeless immigrants* (*n* = 1); *raising awareness of immigrants regarding the UK healthcare system* (*n* = 1); *targeted community outreach activities and drop-ins* (*n* = 1); and *empowerment of Immigrants with regard to health and social determinants* (*n* = 1). Furthermore, 25 items (51%) of the 49 items achieved mean scores of ≥ 4.5 and were thus included in round two of the Delphi survey (see Table 2). Notably, the 2 categories of *targeted community outreach activities and drop-ins* (*n* = 2 items) and *research and epidemiology* (*n* = 2 items) had none of their items achieve a mean score of ≥ 4.5.

**Table 2.** Top 25 ranked strategies following round 1 of the Delphi survey

Item	25 Strategies	Mean (SD)	Important and very important (frequency)
Improving and promoting mental health services among homeless immigrants			
1	There is need to improve diversity of mental health professionals to enable culturally appropriate interactions and improve communication with homeless immigrants.	4.83 (0.39)	100
2	There is need to employ more mental health professionals so as to reduce work overload among mental health professionals.	4.75 (0.45)	100
3	There is need to provide secure accommodation where homeless immigrants can have safe and quality sleep. This can also positively impact their mental well-being.	4.50 (0.67)	91.7
Raising awareness of immigrants regarding the UK healthcare system			
4	There is a need to raise awareness among homeless immigrants on the available primary care services, and how they can be accessed.	4.83 (0.39)	100
5	There is a need to raise awareness among undocumented immigrants about their rights to access primary care services and further reassure them that healthcare providers do not share their information with Home Office.	4.67 (0.49)	100
6	There is a need to raise awareness among homeless immigrants that they can access GP surgeries even if they do not share their home addresses with surgeries.	4.5 (0.52)	100
Fight against discrimination and prejudice and respect differences			
7	There is a need to ensure that GP staff respect, create trust, and treat everybody equally without prejudice regardless of their immigration status or homelessness.	4.83 (0.38)	100
8	There is a need to review and/or develop and enforce policies against all forms of discrimination within the healthcare system.	4.73 (0.47)	100
9	There is a need to ensure that healthcare providers deliver healthcare services without any form of discrimination, such as xenophobia or racism.	4.67 (0.49)	100
10	There is a need to motivate healthcare providers so that they deliver healthcare to homeless immigrants with improved attention to their specific needs and priorities	4.50 (0.52)	100
Addressing the social determinants of health			
11	There is need to provide suitable accommodation to homeless immigrants, for example, that is in a good state, and free of vectors like bedbugs and mice.	4.73 (0.47)	100
12	There is need to provide accommodation for homeless immigrants that require medical treatment in accordance to the human rights approach to care. This applies in situations where a homeless immigrant has a serious healthcare need that warrants accommodation during the course of treatment.	4.50 (0.82)	81.8
Improving GP registration services			
13	There is need to raise awareness among the surgery staff on homeless immigrants’ rights to accessing primary care services. For example, they should be informed that every homeless immigrant has a right to access primary care services regardless of their immigration status.	4.72 (0.47)	100
Enabling access to benefits and financial support			
14	There is a need to raise awareness among healthcare providers on who can access free prescriptions and the required paperwork for such eligibility. This ensures that homeless immigrants have access to free prescriptions.	4.67 (0.49)	100
Provision of culturally sensitive primary care services			
15	There is a need for healthcare providers to receive specific training on cultural competencies and communication skills.	4.67 (0.49)	100
16	There is a need to raise awareness among GP surgeries about homeless immigrants. Since being the gatekeepers to the NHS, surgeries need to understand more about the people who present to them as they come from various communities with varying gender and cultural expectations.	4.58 (0.51)	100
17	There is a need to integrate cross-cultural training into professional development and training activities for health care providers.	4.5 (0.67)	91.7
18	There is a need for health education and health promotion messages to take into account cultural diversity.	4.5 (0.52)	100
Empowerment of immigrants with regard to health and social determinants			
19	There is a need to support homeless immigrants in developing social networks within their communities. For example, through linkages to support groups, organizations, events, community centres, etc.	4.63 (0.50)	100
20	There is a need to raise awareness and educate homeless immigrants about their rights, entitlements, and support (such as benefits) particularly when they are new in the country.	4.5 (0.67)	91.67
Intersectoral collaboration			
21	Increasing the involvement of homeless immigrants and voluntary sector providers in the planning and delivery of primary care services.	4.58 (0.67)	91.7
22	There is a need to put in place measures that ensure that the integrated care system (ICS), which addresses both health and social issues, has a meaningful impact at the community level.	4.50 (0.52)	100
Changes in immigration policies			
23	There is a need to increase opportunities for asylum seekers to engage in formal and informal employment. This ensures their safety against exploitation and that they can afford basic needs and health-related costs such as transport costs, and phone credit, among others.	4.58 (0.67)	91.7
Improving the quality of primary care services			
24	There is a need for health service providers to treat homeless immigrants with respect without stereotyping them based on their immigration status or their homelessness.	4.58 (0.67)	91.7
Improving communication between immigrants and healthcare providers			
25	There is a need to provide high-quality interpreter services, either in person or by telephone. There is also a need to make these services easily accessible to homeless immigrants.	4.5 (0.52)	100

SD = Standard deviation.

In the second round, 10 items with the highest mean scores were chosen as key priority strategies ranked by stakeholders who participated in the study (see Table 3). Four of the top five rated strategies belonged to the two categories of *improving and promoting mental health services among homeless immigrants* and *fighting against discrimination and prejudice, and respecting differences.* Specifically, under *improving and promoting mental health services among homeless immigrants*, the need to improve the diversity of mental health professionals (Mean = 4.58 (SD = 0.49), 100% consensus) and the need to employ more mental health professionals (Mean = 4.58 (SD = 0.67), 91.7% consensus) ranked as 3^rd^ and 4^th^, respectively.

**Table 3.** Top 10 ranked strategies following round 2 of the Delphi survey

Item rank	Top 10 strategies	Mean (SD)	Important and very important (frequency)
Improving and promoting mental health services among homeless immigrants			
4	There is need to employ more mental health professionals so as to reduce work overload among mental health professionals.	4.58 (0.67)	91.7
3	There is need to improve diversity of mental health professionals to enable culturally appropriate interactions and improve communication with homeless immigrants.	4.58 (0.49)	100
Raising awareness of immigrants regarding the UK healthcare system			
7	There is a need to raise awareness among homeless immigrants on the available PCS and how they can be accessed.	4.42 (0.67)	91.7
8	There is a need to raise awareness among undocumented immigrants about their rights to access PCS and further reassure them that healthcare providers do not share their information with Home Office.	4.41 (0.51)	100
Fight against discrimination and prejudice and respect differences			
6	There is a need to motivate healthcare providers so that they deliver healthcare to homeless immigrants with improved attention to their specific needs and priorities	4.42 (0.51)	100
1	There is a need to ensure that GP staff respect, create trust, and treat everybody equally without prejudice regardless of their immigration status or homelessness.	4.75 (0.45)	100
5	There is a need to ensure that healthcare providers deliver healthcare services without any form of discrimination, such as xenophobia or racism.	4.50 (0.52)	100
Empowerment of Immigrants with regard to health and social determinants			
2	There is a need to raise awareness and educate homeless immigrants about their rights, entitlements, and support (such as benefits) particularly when they are new in the country.	4.67 (0.49)	100
Provision of culturally sensitive PCS			
9	There is a need to raise awareness among GP surgeries about homeless immigrants. Since being the gatekeepers to the NHS, surgeries need to understand more about the people who present to them as they come from various communities with varying gender and cultural expectations.	4.33 (0.65)	91.7
10	There is a need for healthcare providers to receive specific training on cultural competencies and communication skills.	4.27 (0.79)	81.8

SD = Standard deviation.

The need to ensure that GP staff respect, create trust, and treat everybody equally without prejudice (Mean = 4.75 (SD = 0.45), 100% consensus) was ranked as 1^st^ and the need to ensure that healthcare providers deliver healthcare services without any form of discrimination (Mean = 4.50 (SD = 0.52), 100% consensus) was ranked as 5^th^. Both items belonged to the category of *fighting against discrimination and prejudice and respecting differences.* The remaining item which was ranked as 2^nd^ belonged to the category *of empowerment of immigrants with regard to health and social determinants.* This item concerned the need to raise awareness and educate homeless immigrants about their rights, entitlements, and support particularly when they are new in the country (Mean = 4.67 (SD = 0.49), 100% consensus). Among the top 10 items, the least ranked items (i.e., 9^th^ and 10^th^) were the need to raise awareness among GP surgeries about homeless immigrants (Mean = 4.33 (SD = 0.65), 91.7% consensus) and the need for healthcare providers to receive specific training on cultural competencies and communication skills (Mean = 4.27 (SD = 0.79), 81.8% consensus). Both items belonged to the category of *provision of culturally sensitive PCS.*

## Discussion

Using a Delphi consensus method, we were able to identify and rank the top 10 priority strategies from an initial list of 58 strategies suggested by homeless immigrants and stakeholders for improving access to PCS for homeless immigrants in England. Based on the list of the top five priority strategies, our study highlights that fighting against discrimination and prejudice and respecting differences; improving and promoting mental health services; and empowering homeless immigrants in regard to their health and social determinants were the three most outstanding intervention areas identified to improve access to PCS for homeless immigrants in Kent and London.

Our study identified the top priority strategy (out of a total of 10) as the need for healthcare providers to demonstrate respect, build trust, and treat everyone equally, regardless of their immigration status or homelessness. This finding is well corroborated by previous studies conducted in England that revealed how some homeless individuals perceived discrimination and stigmatization from both community members and healthcare providers. These experiences were attributed to their living conditions, racial background, immigration status, and health-related issues (Gunner *et al*., 2019). Furthermore, earlier research demonstrated that social segregation significantly eroded trust in public institutions, including the NHS, thereby creating additional obstacles to accessingPCS (Karlsen and Nelson, 2021; *Paul et al.*, 2022). This potentially demonstrates the high level of recognition, readiness, and commitment among stakeholders to counter the issue of discrimination. Based on our research, it is evident that in addition to people with lived experiences, public health initiatives must also involve PCS stakeholders in developing culturally appropriate solutions to address discrimination, instead of adopting a one-size-fits-all approach (Hinkel, 2011). The involvement of PCS stakeholders is important because they have a comprehensive understanding of its enormous existence. One of the top 10 recommendations was the provision of culturally appropriate healthcare, which is a crucial factor in addressing discrimination, despite being ranked last. This reinforces the recognition that discrimination is a significant issue that needs to be addressed. These efforts are crucial for promoting equitable access to healthcare services for marginalized groups, as recommended by other researchers (Skosireva *et al.*, 2014). Overall, curbing discrimination against immigrants and marginalized populations contributes to the preservation and well-being of their health, aligning with the broader objective of enhancing overall health outcomes and reducing disparities in healthcare access (Szaflarski and Bauldry, 2019).

Our findings reveal that the need to improve and promote mental health services for homeless immigrants was of high priority for PCS stakeholders. Related to mental health, we find that the suggested solutions are both applicable to improving the mental health of homeless immigrants, as well as that of the healthcare providers. For instance, stakeholders prioritized the need to address the work overload among mental health professionals by employing more professionals and enhancing diversity within the workforce to facilitate culturally appropriate interactions and improve communication with homeless immigrants. First of all, mental health problems are reported as highly prevalent in homeless (Hossain *et al.*, 2020) and immigrant (Blackmore *et al.*, 2020) populations in the UK and elsewhere (Gil-Salmeron *et al.*, 2021). Yet, mental health services continue to experience overwhelming health staff turnover, underfunding and excessive workloads (Bergman *et al.*, 2021). Research shows that mental health problems among immigrants in the UK have been exacerbated by the strain on healthcare professionals (Pollard and Howard, 2021). Additionally, the COVID-19 pandemic has been shown to have exacerbated the pre-existing mental health inadequacies among health systems and at the same time increased the burden of mental health across populations (Gillard *et al*., 2021). Healthcare workers also experienced work overload, burnout, anxiety, and other mental health issues exacerbated by COVID-19, for example, rapid transition to online service delivery, and fear of contagion, among others (San Juan *et al*., 2021). A report by the UK’s Health and Social Care Committee highlighted chronic excessive workload as a significant contributor to burnout and staff shortages within the NHS, with existing shortages even before the full impact of the pandemic (Health and Social Care Committee, 2021, p.10).

Indeed, the suggestions on mental health raised by our study participants align with findings from other studies that emphasize the importance of increased human and physical resource investment in mental health services, as well as the need for evaluation of existing strategies to improve access and quality of mental healthcare for immigrants (Giacco *et al.*, 2014). Also consistent with our findings, previous research emphasizes the need for the enhancement of diversity among mental health professionals as a crucial step toward the provision of culturally sensitive care to immigrants (Gopalkrishnan and Babacan, 2015). In line with our findings, the NHS mental health implementation plan for 2019/20 – 2023/24 recognizes the need to provide mental health support for the homeless population, through the establishment of a mechanism to assess their needs and provide trauma-informed care with the involvement of multiple delivery partners, as well as targeting the provision of specialist mental health services in the areas where they are most needed (NHS England, 2019, p.42).

We found that the need for strategies to empower homeless immigrants in relation to their health and social determinants emerged as one of the top three intervention areas. Specifically, the key strategy identified within this intervention area was the need to raise awareness of the rights to receive support and access PCS among homeless immigrants. Indeed, various studies identify the lack of awareness of rights and differing interpretations and implementation of rights at regional, institutional, and individual levels as some key barriers to access to PCS among immigrants and homeless people (Woodward *et al.*, 2014; Neves-Silva *et al.*, 2018). This finding emphasizes the key role of adopting a rights-based approach in PCS delivery. Similar to our findings, the increased recognition of the urgency for a rights-based approach to tackling homelessness in many parts of the developed world has been documented (Fitzpatrick and Pleace, 2012; Kenna and Fernandez Evangelista, 2013). The rights-based approach was created as a means to operationalize and expand human rights, based on the notion that it’s a first step towards the empowerment of excluded groups by acknowledging that those individuals have rights (Kenna and Fernandez Evangelista, 2013).

In our study, the four lowest ranked among the 10 strategies belonged to the intervention areas of raising awareness of immigrants regarding the UK healthcare system and the provision of culturally sensitive PCS. Although ranked the lowest among the top 10, these intervention areas and strategies remain crucial for enhancing access to PCS for homeless immigrants. In line with these two broad intervention areas, the co-design of health interventions has been coined as one of the effective participatory approaches for empowering vulnerable sub-populations to take charge of their health and livelihood (Cheng *et al.*, 2021). There is research demonstrating improvement in health and livelihood through having healthcare professionals or social workers working alongside immigrants or homeless people to co-design well-tailored and culturally appropriate health interventions and informational materials addressing important topics, for example, on the operation of the healthcare system, on targeted services and offers (e.g. legal privileges, financial aid, subsidies, food, and medical supplies), and available community level resources or supports (Keygnaert *et al.*, 2013; Wong *et al.*, 2014). Previous research has underscored the significant barriers created by the absence of culturally competent services in immigrants’ access to healthcare, particularly when seeking assistance for healthcare issues where cultural variations make it challenging for providers to comprehend the causes and experiences of illness (Wood and Newbold, 2012). Researchers have emphasized the significance of understanding the cultural needs of individuals seeking treatment and advocating for policies that ensure inclusivity based on specific cultural affiliations (Giacco *et al.*, 2014; Sarkar and Punnoose, 2017). Evidence from a previous systematic review shows that cultural competence training is an effective intervention that can enable healthcare providers to provide culturally competent care that increases the satisfaction of patients from minority groups (Govere and Govere, 2016). Research also shows that it is important to ensure that the health workforce comprises inter-professional teams who can help to bring distinct skills, training, and prior patient experiences as this is shown to uniquely address varied individual needs and expectations, thus improving PCS quality (McGregor *et al.*, 2019).

### Implications for policy and practice

The findings of this study have important implications for policy and practice. Implementing these top ten priority strategies has the potential to enhance access to healthcare and improve health outcomes for homeless immigrants (Parker *et al.*, 2021). These identified priorities reflect the valuable perspectives of healthcare providers on how to address the issue of improving access to PCS for homeless immigrants. Consequently, the prioritized list serves as recommendations to enhance the healthcare system in the UK by integrating these strategies into primary care practices. The implications of these findings highlight the need for policy changes and practice guidelines that align with the identified priority strategies. The current findings on the prioritized strategies for improving access to PCS for homeless immigrants are highly relevant for post-COVID recovery in terms of improving their healthcare. The pandemic exacerbated existing health inequalities, including access to healthcare for marginalized populations. By implementing the identified strategies for improving access to PCS, healthcare providers can better serve homeless immigrants and improve their health outcomes. Therefore, policymakers and healthcare organizations should consider the study’s recommendations to develop interventions and initiatives aimed at improving access to PCS for homeless immigrants, particularly in the context of post-pandemic recovery efforts. Additionally, the identified areas for intervention provide a platform for advocacy and lobbying, especially by healthcare providers of marginalized populations like homeless immigrants. These are possibly insufficiently funded sub-sectors and any lobbying goes a long way in improving resource allocation. The findings are highly informative in supporting evidence-informed decision-making (EIDM) considering that research on this subject has been scarce and further constrained by COVID-19. Future research should focus on evaluating the effectiveness of the identified priority strategies in improving access to PCS for homeless immigrants. Various studies including longitudinal studies, larger feasibility studies, community trials, or comparative studies can be conducted to determine whether the implementation of the recommended interventions leads to sustained improvements in health outcomes for this population. Furthermore, research can focus on identifying priority strategies from the perspectives of homeless immigrants themselves to gain a deeper understanding of their healthcare needs and preferences. Additionally, healthcare providers should be encouraged to receive training and education on cultural competence, mental health support, and addressing discrimination to effectively implement these strategies in their daily practice.

Furthermore, there is a need for improved holistic monitoring of the performance and quality of PCS, with a stronger emphasis on assessing indicators of social justice and equity within service delivery. This enhancement would facilitate the evaluation of disparities between more and less advantaged social groups. Given that the purpose of monitoring is to provide information that guides policies and programs towards promoting greater health equity, an effective monitoring system should encompass the additional steps required to disseminate findings to policy-makers, program managers, and organizations that represent stakeholders capable of leveraging this information to foster increased equity in service provision (Braveman, 2003). By incorporating these strategies into policy and practice, we can work towards creating a more inclusive and accessible healthcare system for homeless immigrants in England.

### Strength and limitations

Our study exhibits several strengths and limitations that should be taken into consideration. A notable strength is the inclusion of diverse stakeholders such as healthcare providers and voluntary sector providers who have experience supporting immigrants and homeless individuals in the UK. This engagement of a wide range of stakeholders has been found to enhance the perceived relevance and adoption of research findings by health systems (Concannon *et al*., 2012). Another strength was the utilization of an online survey during the Delphi rounds, enabling participants to provide evaluations regardless of their geographical location and to address barriers in communication and research posed by the COVID-19 pandemic. Furthermore, using the Delphi approach provided a more systematic approach to identifying the top priority suggestions and helped to move beyond a mere list of suggestions but rather a further consensus towards the most prioritized suggestions (Niederberger and Spranger, 2020). However, there are certain limitations that need to be acknowledged. One limitation is the exclusion of homeless immigrants from the Delphi study due to challenges related to digital connectivity. Yet, incorporating the perspectives of homeless immigrants would have provided valuable insights into the topic under investigation. Although the Delphi technique lacks universally agreed-upon criteria or a specific number for expert selection, in this study, the limited participation of only two voluntary sector providers and the absence of local council professionals may have impacted the comprehensiveness and diversity of the collected results (Keeney *et al*., 2006). Additionally, our study had a low response rate (55%) which could have impacted the quality of our data to some extent. Nonetheless, we prioritized representativeness by including a diverse range of healthcare providers in our panel composition. Additionally, due to the small sample sizes within specific subgroups, such as voluntary sector providers, we were unable to conduct a subgroup analysis of item rankings. Lastly, we did not collect data related to the years of work experience of the stakeholders. We acknowledge that without this information, it might become challenging to assess the level of expertise and depth of knowledge possessed by the participants.

## Conclusion

Our Delphi consensus method, involving stakeholders experienced in supporting immigrants and homeless individuals, successfully identified priority strategies for enhancing access to PCS for homeless immigrants. The top three strategies identified encompassed fighting against discrimination and prejudice, improving and promoting mental health services, and empowering homeless immigrants. These evidence-based strategies hold the potential to support the implementation of healthcare interventions aimed at improving access to PCS and healthcare outcomes for homeless immigrants. However, it is crucial to conduct further research that includes homeless immigrants using the Delphi approach or other consensus approaches to gain insights into the strategies that are most important to them in enhancing access to PCS, as they are the primary target users. Such research will contribute to the development of comprehensive and effective interventions tailored to the specific needs and experiences of homeless immigrants.

## Supplementary material

For supplementary material accompanying this paper visit http://doi.org/10.1017/S1463423623000646. click here to view supplementary material
