# Supplementary material for: Strategies for improving access to primary care services for homeless immigrants in England: a Delphi study
Source: Prim Health Care Res Dev. 2023 Dec 14;24:e70. doi: 10.1017/S1463423623000646 (PMC10790723; doi:10.1017/S1463423623000646)
Supplement: Supplementary file 1 [file S1463423623000646sup001.docx]

**Supplementary File 1: Mean scores and frequencies of 58 items following round 1 of the Delphi survey**

| Item | Strategies | Mean scores (Standard deviation) | Important & Very Important (%) | Consensus achieved ≥75%  (Yes/No) |
| --- | --- | --- | --- | --- |
| Improving communication between immigrants and healthcare providers | |  |  |  |
| 01 | Training the GP administration and reception staff to clearly inform immigrants what kind of interpretation and translation services they can access and how they can access them. | 4.08 (0.90) | 66.7 | No |
| 02 | Awareness raising for GP staff on the entitlements of immigrants to access interpreters. | 4.25 (0.97) | 83.3 | Yes |
| 03 | There is a need to provide high-quality interpreter services, either in person or by telephone. There is also a need to make these services easily accessible to homeless immigrants. | 4.5 (0.52) | 100.0 | Yes |
| 04 | Translation of leaflets giving basic information about health services in some of the languages of the immigrants. | 4 (1.04) | 66.7 | No |
|  |  |  |  |  |
| Enabling access to benefits and financial support | |  |  |  |
| 05 | There is need to raising awareness of healthcare providers on who can access free prescriptions and the required paperwork for such eligibility. This ensures that homeless immigrants have access to free prescriptions. | 4.67 (0.49) | 100.0 | Yes |
|  |  |  |  |  |
| Provision of culturally sensitive primary care services | |  |  |  |
| 06 | There is a need to raise awareness among GP surgeries about homeless immigrants. Since being the gatekeepers to the NHS, surgeries need to understand more about the people who present to them as they come from various communities with varying gender and cultural expectations. | 4.58 (0.51) | 100.0 | Yes |
| 07 | Co-design services with homeless immigrants to ensure the services provided are tailored to respond to the healthcare needs of this population group. | 4.42 (0.79) | 83.3 | Yes |
| 08 | There is a need for healthcare providers to receive specific training on cultural competencies and communication skills. | 4.67 (0.49) | 100.0 | Yes |
| 09 | Employ cultural mediators or healthcare providers of migrant descent. | 3.5 (0.67) | 41.7 | No |
| 10 | There is a need for health education and health promotion messages to take into account cultural diversity. | 4.5 (0.52) | 100.0 | Yes |
| 11 | There is a need to integrate cross-cultural training into professional development and training activities for health care providers. | 4.5 (0.67) | 91.7 | Yes |
|  |  |  |  |  |
| Improving and promoting mental health services among homeless immigrants | |  |  |  |
| 12 | Establishing Bespoke services based around social activities such as football groups rather than the formal Bespoke Cognitive Behavioural Therapy (CBT) and ensuring that homeless immigrants have access to information about these services through their social workers. | 4.25 (0.96) | 83.3 | Yes |
| 13 | Offering mental health assessment and support to the newly arrived immigrants in the country to support them to process the trauma that they might have experienced. | 4.25 (0.96) | 91.7 | Yes |
| 14 | Establishing music as a therapeutic service for homeless immigrants with language difficulties to enable them to express themselves without having the need to have proficient language skills. | 3.42 (1.16) | 41.7 | No |
| 15 | There is need to provide secure accommodation where homeless immigrants can have safe and quality sleep. This can also positively impact their mental wellbeing. | 4.50 (0.67) | 91.7 | Yes |
| 16 | There is need to employ more mental health professionals so as to reduce work overload among mental health professionals. | 4.75 (0.45) | 100.0 | Yes |
| 17 | There is need to improve diversity of mental health professionals to enable culturally appropriate interactions and improve communication with homeless immigrants | 4.83 (0.39) | 100.0 | Yes |
|  |  |  |  |  |
| Improving GP registration services | |  |  |  |
| 18 | There is need to raise awareness among the surgery staff on homeless immigrants’ rights to accessing primary care services. For example, they should be informed that every homeless immigrant has a right to access primary care services regardless of their immigration status. | 4.72 (0.47) | 100.0 | Yes |
| 19 | The surgery receptionists should work in hand with the social prescribers to support homeless immigrants without an address to register. | 4.33 (0.89) | 100.0 | Yes |
| 20 | Social prescribers in surgeries should guide homeless immigrants to register with the GP surgery. | 3.91 (1.04) | 100.0 | Yes |
|  |  |  |  |  |
| Addressing the social determinants of health | |  |  |  |
| 21 | Social prescribers should follow up on individuals who are socially isolated by speaking to them on phone now and again. | 4.42 (0.67) | 100.0 | Yes |
| 22 | Social prescribers should support homeless immigrants with difficulties in accessing housing and other benefits to fill out forms and apply for benefits, and/or signpost them to organizations that can help them fill out these forms and navigate the system. | 4.08 (0.99) | 75.0 | Yes |
| 23 | Ensuring a wide coverage of social prescribers across Kent and Medway. | 4.42 (0.67) | 100.0 | Yes |
| 24 | Provision of mobile transportation, to and from surgeries, to support homeless immigrants who cannot afford transport costs to access surgeries. | 4.08 (0.67) | 83.3 | Yes |
| 25 | There is need to provide accommodation for homeless immigrants that require medical treatment in accordance to the human rights approach to care. This applies in situations where a homeless immigrant has a serious healthcare need that warrants accommodation during the course of treatment. | 4.50 (0.82) | 81.8 | Yes |
| 26 | There is need to provide suitable accommodation to homeless immigrants, for example, that is in a good state, and free of vectors like bedbugs and mice. | 4.73 (0.47) | 100.0 | Yes |
|  |  |  |  |  |
| Intersectoral collaboration | |  |  |  |
| 27 | Coordinated actions between the health and social sectors so that services wrap around the person rather than the person having to navigate their way around different services because often, if homeless immigrants haven't got permanent housing, it’s hard to access a GP surgery. | 4.42 (0.79) | 83.3 | Yes |
| 28 | There is a need to put in place measures that ensure that the Integrated Care System (ICS) which addresses both health and social issues, has a meaningful impact at the community level. | 4.50 (0.52) | 100.0 | Yes |
| 29 | Increasing the involvement of homeless immigrants and voluntary sector providers in the planning and delivery of primary care services. | 4.58 (0.67) | 91.7 | Yes |
|  |  |  |  |  |
| Raising awareness of immigrants regarding the UK healthcare system | |  |  |  |
| 30 | There is a need to raise awareness among homeless immigrants on the available primary care services, and how they can be accessed | 4.83 (0.39) | 100.0 | Yes |
| 31 | Raising awareness of immigrants about waiting times for appointments because it might not be a racist thing to be on the waiting list. | 3.83 (1.03) | 58.3 | No |
| 32 | There is a need to raise awareness among undocumented immigrants about their rights to access primary care services and further reassure them that healthcare providers do not share their information with Home Office. | 4.67 (0.49) | 100.0 | Yes |
| 33 | Provision of special consultation the first-time homeless immigrants access primary care services. | 3.83 (0.94) | 100.0 | Yes |
| 34 | There is a need to raise awareness among homeless immigrants that they can access GP surgeries even if they don’t share their home addresses with surgeries. | 4.5 (0.52) | 100.0 | Yes |
|  |  |  |  |  |
| Changes in immigration policies | |  |  |  |
| 35 | There is a need to increase opportunities for asylum seekers to engage in formal and informal employment. This ensures their safety against exploitation and that they can afford basic needs and health-related costs such as transport costs, and phone credit, among others. | 4.58 (0.67) | 91.7 | Yes |
| 36 | Provision of accommodation to homeless immigrants without recourse to public funds. | 4.0 (0.85) | 100.0 | Yes |
| 37 | NHS charges for secondary care should be reduced for immigrants who cannot make ends meet. | 4.25 (0.75) | 83.3 | Yes |
|  |  |  |  |  |
| Targeted community outreach activities and drop-ins | |  |  |  |
| 38 | Carrying out more community outreaches to create visibility of healthcare workers in areas with high migrant populations to enable homeless immigrants who might find it challenging to ask for help to know that healthcare workers are available on the ground and can support them. | 4.42 (0.51) | 100.0 | Yes |
| 39 | Establishment of drop-ins for homeless immigrants at the surgeries outside the normal days of Mon-Fri and hours of 9-5pm to ensure flexibility of healthcare services so that homeless immigrants can access these services. | 4.0 (1.18) | 72.7 | No |
|  |  |  |  |  |
| Improving the quality of primary care services | |  |  |  |
| 40 | Provision of reception services for homeless immigrants who might experience digital exclusion to be able to walk into surgeries and access services like registration and booking appointments. So those who can use digital can do so, as others who can’t are helped via the reception. | 4.17 (0.83) | 75.0 | Yes |
| 41 | There is a need for health service providers to treat homeless immigrants with respect without stereotyping them based on their immigration status or their homelessness. | 4.58 (0.67) | 91.7 | Yes |
| 42 | Surgeries should consider the individuals’ specific medical history and social background and give individualised psychological support and empathy. | 4.42 (0.79) | 83.3 | Yes |
| 43 | Healthcare professionals should take the time to listen to homeless immigrants and check that both parties have understood each other. | 4.45 (0.69) | 90.9 | Yes |
| 44 | Provision of extra consultation time when dealing with homeless immigrant patients to allow for interpretation and to gain an understanding of a new culture. | 4.36 (0.81) | 81.8 | Yes |
| 45 | Allocation of GP slots to homeless immigrants because at the moment, homeless immigrants are not considered vulnerable and not given priority hence they have got the same treatment as everyone else such as making long queues that hinders some from registering with surgeries. | 3.67 (1.07) | 58.3 | No |
| 46 | Developing protocols on how to provide person-centred care to homeless immigrants and ensuring that surgeries have access to these protocols and that they know what to do especially in surgeries that might not have many immigrant populations coming in frequently. | 4.45 (0.69) | 90.9 | Yes |
| 47 | Surgeries should shorten the waiting times for GP appointments. | 3.92 (0.90) | 58.3 | No |
| 48 | Government should provide additional payments to health practitioners at surgeries to reflect the additional time given to immigrants so that they're more likely to get the health care that they need. Otherwise, it's altruistic on the part of the GPs to take on people yet they're not going to get paid for the amount of work that they have to put in. | 4.17 (0.83) | 75.0 | Yes |
|  |  |  |  |  |
| Empowerment of Immigrants with regard to health and social determinants | |  |  |  |
| 49 | Provision of information for migrants in their own language about their rights and the functioning of the UK health care system and social care system. | 4.25 (0.87) | 75.0 | Yes |
| 50 | Provision of opportunities to learn English to facilitate integration into the UK system and consequently facilitate access to health care. | 4.25 (0.97) | 66.7 | No |
| 51 | There is a need to support homeless immigrants in developing social networks within their communities. For example, through linkages to support groups, organisations, events, community centres, etc. | 4.63 (0.50) | 100.0 | Yes |
| 52 | There is a need to raise awareness and educate homeless immigrants about their rights, entitlements, and support (such as benefits) particularly when they are new in the country. | 4.5 (0.67) | 91.67 | Yes |
|  |  |  |  |  |
| Fight against discrimination and prejudice, and respect differences | |  |  |  |
| 53 | There is a need to ensure that GP staff respect, create trust, and treat everybody equally without prejudice regardless of their immigration status or homelessness. | 4.83 (0.38) | 100.0 | Yes |
| 54 | There is a need to ensure that healthcare providers deliver healthcare services without any form of discrimination, such as xenophobia or racism. | 4.67 (0.49) | 100.0 | Yes |
| 55 | There is a need to motivate healthcare providers so that they deliver healthcare to homeless immigrants with improved attention to their specific needs and priorities. | 4.50 (0.52) | 100.0 | Yes |
| 56 | There is a need to review and/or develop and enforce policies against all forms of discrimination within the healthcare system. | 4.73 (0.47) | 100.0 | Yes |
|  |  |  |  |  |
| Research and epidemiology | |  |  |  |
| 57 | Health care services should be provided with relevant knowledge on health and risk factors concerning the populations they are dealing with. | 4.25 (0.75) | 83.3 | Yes |
| 58 | Healthcare registries should record and monitor migrant health to facilitate migrant health research. | 4.33 (0.65) | 91.7 | Yes |
